# Why the Sea Is Green

**Published:** 1890-04

**Authors:** 


### WHY THE SEA IS GREEN.

The green color of ocean water depends upon the number of medusæ and other minute animal forms which inhabit it. The deep green Northern seas literally swarm with these miniature creatures; in some places as many as 128 of them have been found in a single cubic inch of water. In this proportion a cubic foot of water would contain 221,184; a cubic fathom, 47,776,744, and a cubic mile 48,776,000,000,000. From soundings made in the vicinity of where these creatures are found in such immense numbers, it is probable that the waters will average a mile in depth; whether these forms occupy the whole depth is uncertain. Provided, however, that the depth to which they extend is but 250 fathoms, the above immense number of one species may occur within a space of one square mile. It may give a better conception of the immense number of medusæ in this extent, if we calculate the length of time that would be requisite for a certain number of persons to count this number. Allowing that one person count 1,000,000 in seven days, which is barely possible, even at this rapid rate it would have been necessary for 80,000 persons to have commenced counting at the time of Adam in order to complete the enumeration in time for the census of 1890.

What a stupendous idea does this fact give of the immensity of creation ! But if the number of these little living things in a space of one single mile be so great, what must be the number required for discoloring the hundreds of thousands of miles contained in the oceans of the globe.

